# Effects of tanshinol on markers of bone turnover in ovariectomized rats and osteoblast cultures

**DOI:** 10.1371/journal.pone.0181175

**Published:** 2017-07-26

**Authors:** Jianfeng Han, Wei Wang

**Affiliations:** 1 Department of Orthopedics, The 2nd Affiliated Hospital of Harbin Medical University, Harbin, China; 2 Department of Endocrinology, The 2nd Affiliated Hospital of Harbin Medical University, Harbin, China; University of Massachusetts Medical School, UNITED STATES

## Abstract

This study was aimed to explore the role of tanshinol in osteoblastic cells, and the role *in vivo* using an ovariectomized (OVX) rat model of osteoporosis. MC3T3-E1 cells were pretreated with 0–400 μg/mL tanshinol, and then cell viability, apoptosis, alkaline phosphatase (ALP) activity and the expressions of Collagen Type I Alpha 1 (Col1A1), Runt Related Transcription Factor 2 (Runx2) and osteocalcin (OCN) were respectively detected. Rats underwent OVX surgery was intervened with 5 mg/kg tanshinol or 25 μg/kg β-estradiol (E2) for 12 weeks. The triglycerides (TG), total cholesterol (TC), high and low density lipoprotein cholesterol (HDL-C and LDL-C), ALP, OCN and Tartrate-resistant acid phosphatase-5b (TRACP-5b) contents were measured. Besides, the expressions of main factors in nuclear factor-kappa B (NF-κB) pathway were detected. The results showed that tanshinol significantly promoted MC3T3-E1 cells viability and ALP activity, while inhibited apoptosis (*P* < 0.05); Col1A1, Runx2 and OCN were all up-regulated by tanshinol (*P* < 0.05). In OVX rats, the contents of TG, TC, LDL-C, ALP, OCN and TRACP-5b were all increased (*P* < 0.05), while HDL-C was decreased (*P* < 0.05). Tanshinol significantly alleviated these aberrant regulations (*P* < 0.05). Inhibitory subunit of NF-κB (IκBα) and p65 were both remarkably phosphorylated by OVX, while this phosphorylation was partially neutralized by tanshinol (*P* < 0.05). In conclusion, we demonstrated that tanshinol exerted a bone-protective function by modulating the markers of bone turnover possibly via blocking NF-κB pathway. This study will provide new evidence that tanshinol is a potential therapeutic option for the relief of estrogen deficiency-induced osteoporosis.

## Introduction

Osteoporosis is one type of bone metabolic disease characterized by low bone mineral density and deterioration of the bone microarchitecture [1]. It is an increasingly important health problem which affects millions of people worldwide with significant impact on morbidity, mortality, quality of life and cost [2]. The most important risk factors of osteoporosis are advanced age and female sex, and estrogen deficiency following menopause or ovariectomized (OVX) surgery is correlated with a rapid reduction in bone mineral density [3]. In the bone, osteoblasts are responsible for bone formation while the duty of osteoclasts is bone resorption, that is, bone growth is maintained by the coordination of osteoblasts and osteoclasts [4]. Osteoblast differentiation, an important process for its function, confers marked rigidity and strength to the bone while still maintaining some degree of elasticity [5]. Thus, it is beneficial for osteoporosis prevention and treatment to investigate how to promote osteoblast differentiation and increase bone mass.

Tanshinol, 3-(3,4-Dihydroxyphenyl)-2-hydroxypropanoic acid, also known as danshensu is a water-soluble components of *Salvia miltiorrhiza* Bunge [6]. Tanshinol is a polyphenolic compound with two phenolic hydroxyl groups, and because of this it has been identified as an effective natural product antioxidant [7]. In China, it is widely used in traditional medicine for myocardial infarction, coronary heart disease, atherosclerosis, hypertension, hyperlipoidemia, thrombopoiesis and acute ischemic stroke. In terms of osteoporosis, previous studies have indicated tanshinol stimulated bone formation and attenuated dexamethasone-induced inhibition of osteogenesis in larval zebrafish [6]. Additionally, *in vitro* investigation has provided evidences that tanshinol could antagonize glucocorticoids-induced osteoporosis by controlling osteoblast apoptosis [8]. However, the influence of tanshinol on osteoblastic differentiation and OVX-induced osteoporosis has not been exhaustively investigated.

In the present study, mouse osteoblastic cell line MC3T3-E1 was used and pretreated with tanshinol, to explore the role of tanshinol in osteoblastic cells. Moreover, female rats underwent OVX surgery and tanshinol intervention were used to test whether tanshinol has functional effects on OVX-induced dyslipidemia and bone turnover *in vivo*. Furthermore, the main factors in nuclear factor-kappa B (NF-κB) pathway were detected to obtain a possible understanding of tanshinol in OVX-induced osteoporosis deepen into the molecular mechanism level.

## Materials and methods

### Cell culture and tanshinol treatment

Mouse osteoblastic cell line MC3T3-E1 was purchased from the American Type Culture Collection (ATCC; Manassas, VA). Cells were cultured in α-MEM medium (Gibco, Grand Island, NY) supplemented with 10% fetal bovine serum (FBS; Hyclone, Logan, UT, USA), 100 U/mL penicillin and 100 U/mL streptomycin (Sigma-Aldrich, St. Louis, MO), and were maintained at 37°C in a humidified atmosphere containing 5% CO_2_ [9].

**Fig 1:**
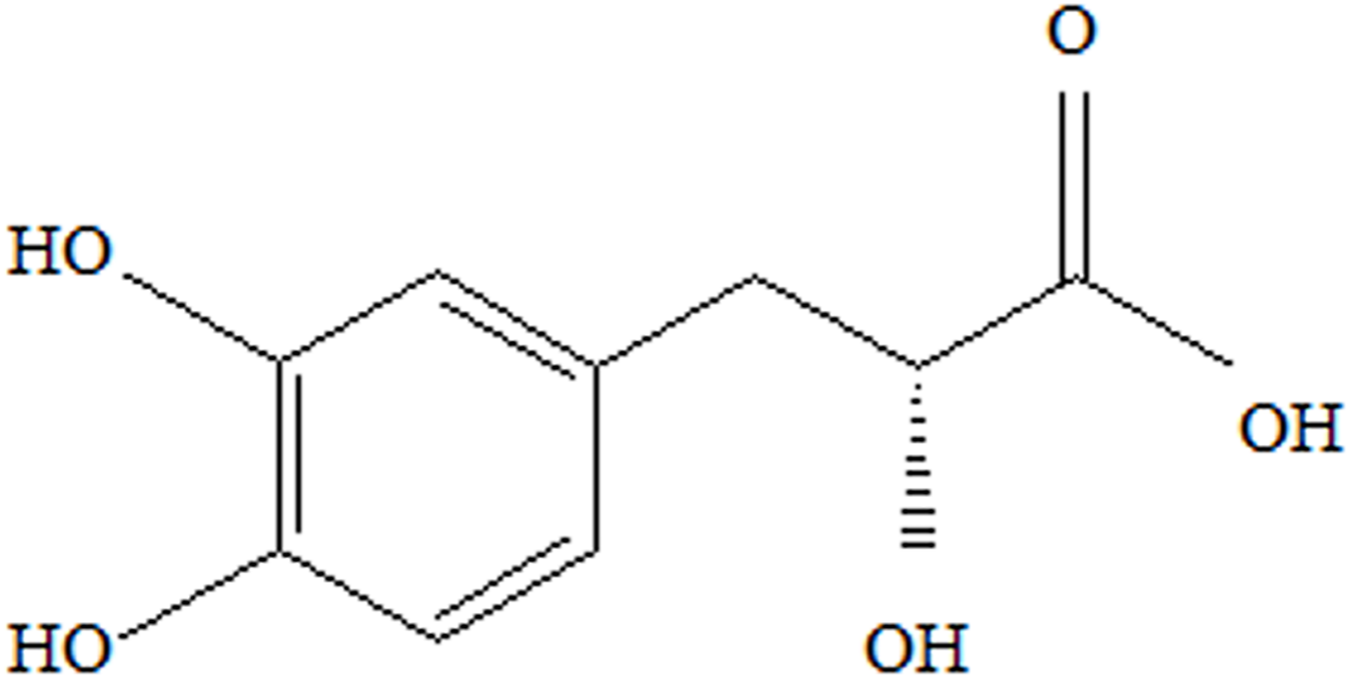
The chemical structure of tanshinol.

Over 99.0% purity of tanshinol was obtained from Tong Ren Tang Company (Beijing, China). The chemical structure of tanshinol was shown in **Fig 1**. The cells were pre-treated with 0–400 μg/mL concentrations of tanshinol for 48 h.

### Cell viability assay

Cell viability was measured by 3-(4, 5-dimethylthiazol-2-yl)-2, 5-diphenyltetrazolium bromide (MTT) assay. Briefly, MC3T3-E1 cells were seeded into 96-well plates at a density of 1 × 10^3^ cells/well and incubated for 24 h. After 48 h incubation of 0–400 μg/mL tanshinol, 20 μL MTT (5 mg/mL; Sigma-Aldrich) was added into each well and the plates were incubated at 37˚C for 4 h. Then, 150 μL dimethyl sulfoxide (DMSO; Sigma-Aldrich) was added to dissolve formazan crystals. The absorbance was measured under a microplate reader (Bio-rod, Hercules, CA) at 570 nm.

### Apoptosis assay

Cell apoptosis was detected by the Annexin V-FITC/PI Kit (4A Biotech Co. Ltd., Beijing, China; Cat. No.: FXP018-100), according to the manufacturer’s protocol. Cells were seeded into 6-well plates at a density 1 × 10^5^ cells/well and treated with 0–400 μg/mL tanshinol for 48 h. Cells were then collected and re-suspended in 200 μL binding buffer containing 10 μL Annexin V-FITC and 5 μL PI with an incubation in the dark for 30 min. Finally, the apoptotic cells were distinguished by a FACS Calibur flow cytometer (Becton-Dickinson) immediately.

### Alkaline phosphatase (ALP) activity

ALP activity was determined by the SensoLyte pNPP ALP Assay Kit (AnaSpec, Fremont, CA, USA; Cat. No.: AS-72146) according to the manufacturer’s protocol. Cells were seeded on the 24-well plates at a density of 2 × 10^4^ cells/well and treated with 0–400 μg/mL tanshinol for 4 days. Afterward, 0.2% Triton X-100 lysis buffer was used to lyses cells, and the optical density of the supernatant of the lysate was measured at 520 nm. The ALP activity was normalizing to the total protein content.

### Real-time quantitative PCR (qPCR)

Cells were first treated with 0–400 μg/mL tanshinol for 48 h, and the total RNA in cells were extracted by using Trizol reagent (Invitrogen, Carlsbad, CA; Cat. No.: 15596–018). Transcriptor First Strand cDNA Synthesis Kit (Roche, Basel, Switzerland; Cat. No.: 04896866001) and 500 ng RNA were used to synthesize cDNA. Real-time qPCR was performed by using FastSTART Universal SYBR Green Master (ROX; Roche, USA; Cat. No.: 04913850001), according to the instructions of manufacture, and conduced on the PCR System 7500 (Applied Biosystems, Foster City, CA, USA). Data were normalized to Glyceraldehyde-3-Phosphate Dehydrogenase (GAPDH) expression, and were analyzed using the classic 2^-△△Ct^ method [10]. All primers were synthesized by GenePharma (Shanghai, China).

### OVX surgery on rats and tanshinol treatment

A total of 40 three-month-old female Sprague-Dawley rats (191–220 g in weight) obtained from Animal Center of the Academy of Military Science of the Chinese PLA (Beijing, China) were employed in this study. Animals were bred and housed under standard conditions as previous described [11]. Experiments using rats were performed in accordance with the National Institute of Health Guide for the Care and Use of Laboratory Animals and were approved by Institutional Animal Care and User Committee of the 2nd Affiliated Hospital of Harbin Medical University. Precautions were taken to minimize suffering and the number of animals used in each experiment.

Rats were randomly allotted into 4 groups (n = 10 in each group) and namely sham, OVX, OVX + E2, and OVX + tanshinol groups. For rats in OVX group, both ovaries were excised under 2% isoflurane anesthesia (Yuyan Instruments, Shanghai, China), according to the technique described previously [12]. The rats in sham group were undergone an incision and suturing without ovary removal. The rats in OVX + E2 and OVX + tanshinol group were first undergone OVX surgery and were respectively treated with 25 μg/kg β-estradiol (E2) [13] and 5 mg/kg tanshinol via oral gavage, started 2 weeks after the surgery and lasted 12 weeks. The rats in sham and OVX group were treated daily with corn oil (Luhua Group, Shandong, China).

Rats were sacrificed by cervical vertebra dislocation to collect the blood samples and femurs.

### Serum lipid analysis

Blood samples from the rats in the four groups were collected and the serum triglycerides (TG), total cholesterol (TC), high density lipoprotein cholesterol (HDL-C) were determined by using diagnostic kits (Asan Pharmaceuticals, Hwasung, Korea; Cat. No.: AM157S-K, AM202-K and AM203-K) on an automatic analyzer (Abbott, model Alcyon 300, USA). Low density lipoprotein cholesterol (LDL-C) was calculated as follows: LDL-C = TC—HDL-C—TG/5.

### Analysis of bone markers

Serum ALP activity was determined by SensoLyte pNPP ALP Assay Kit (AnaSpec; Cat. No.: AS-72146). Both carboxylated and decarboxylated rat serum osteocalcin (OCN) and serum bone-specific ALP concentrations were determined using a rat-specific radioimmunoassay (EIA) kit (Biomedical Technologies, Stoughton, MA, USA; Cat. No.: BT-490) and a commercially available ELISA kit (QUIDEL, San Diego, CA, USA; Cat. No.: 8012). Tartrate-resistant acid phosphatase-5b (TRACP-5b) concentration was determined using a commercial kit (Kamiya Biochemical Company, Seattle, WA, USA; Cat. No.: KT-008) according to the manufacturer’s instructions [14].

### Western blot analysis

Rat femurs were excised and all muscles and connective tissue was removed. The femoral neck were immediately frozen in liquid nitrogen and stored at -80°C until used. Bone protein was extracted from powdered metaphysis and diaphysis as previously described [15]. Cellular proteins in MC3T3-E1 cells were isolated by lysis buffer (Beyotime, Shanghai, China). Protein concentration was determined by Bicinchoninic Acid (BCA) Kit (Thermo Scientific^®^, Rockford, IL; Cat. No.:23225). Proteins were resolved over sodium dodecyl sulfate-polyacrylamide gel electrophoresis (SDS-PAGE) and transferred to nitrocellulose membranes (Merck Millipore, Billerica, MA). After blocked with 5% non-fat milk (BD Sciences, CA) for 1 h, the membranes were incubated with primary antibodies: BCL2 Associated X Protein (Bax; Cat. No.: sc-20067), B-Cell CLL/Lymphoma 2 (Bcl-2; Cat. No.: sc-509), Collagen Type I Alpha 1 (Col1A1; Cat. No.: sc-293182), Runt Related Transcription Factor 2 (RunX2; Cat. No.: sc-390715), OCN (Cat. No.: sc-376835; Santa Cruz Biotechnology, Santa Cruz, CA), inhibitory subunit of NF-κB (IκBα; Cat. No.: ab32518), phosphorylated IκBa (p-IκBa; Cat. No.: ab92700), p65 (Cat. No.: ab16502), phosphorylated p65 (p-p65; Cat. No.: ab86299) and GAPDH (Cat. No.: ab8245; Abcam, Cambridge, MA), at 4°C overnight. The membranes were then incubated in the secondary antibodies for (Cat. No.: ab131368, and ab191866; Abcam) 1 h and the blots were visualized by using enhanced chemiluminescence (ECL) reagent (GE Healthcare, Little Chalfont, UK).

### Statistical analysis

For quantitative data, all results were expressed as the mean ± standard derivations (SD) from at least three independent experiments. Statistical analysis was performed by using the SPSS version 13.0 software (SPSS Inc., Chicago,IL, USA). Different significance between two groups was analyzed by Student’s *t* test; while between three or more groups, different significance was calculated by one-way analysis of variance (ANOVA) with LDS (L) procedure. A P-value < 0.05 was considered statistically significant.

## Results and discussion

### Tanshinol promoted osteoblast viability and ALP activity while reduced apoptosis

**Fig 2:**
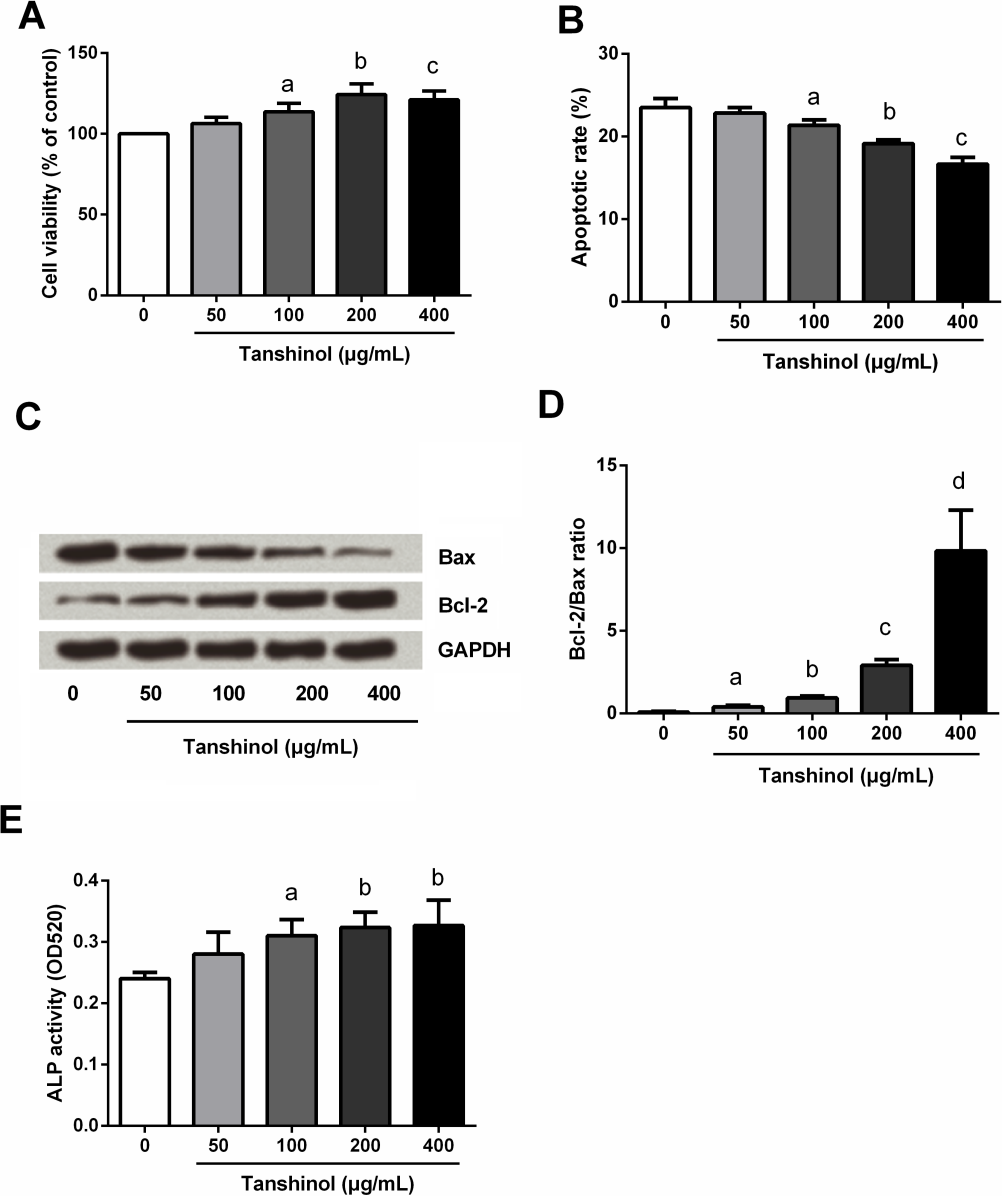
Tanshinol promoted osteoblast viability and ALP activity while reduced apoptosis. MC3T3-E1 cells were treated with 0–400 μg/mL tanshinol, and then (A) cell viability, (B) apoptotic cell rate, (C and D) Bax and Bcl-2 levels, and (E) ALP activity were respectively detected by MTT, flow cytometry, Western blot analysis and ALP assay kit. ALP, alkaline phosphatase; Bax, BCL2 Associated X Protein; Bcl-2, B-Cell CLL/Lymphoma 2; GAPDH, Glyceraldehyde-3-Phosphate Dehydrogenase; MTT, 3-(4, 5-dimethylthiazol-2-yl)-2, 5-diphenyltetrazolium bromide. n = 3. Different lowercase letters above the columns indicate that the mean values of different groups are significantly different (*P* < 0.05).

To explore the functional effects of tanshinol on osteoblast, MC3T3-E1 cells were treated with 0–400 μg/mL tanshinol, and then cell viability, apoptosis and ALP activity were respectively detected by MTT, flow cytometry, Western blot analysis and ALP assay kit. Results in **Fig 2A, 2B** and **2E** showed that, cells treated with 100, 200, and 400 μg/mL of tanshinol possessed higher cell viability and ALP activity, and possessed lower apoptotic cell rate than the control cells without tanshinol treatment (*P* < 0.05). There was no significant change in cell viability, apoptotic cell rate and ALP activity were found in cells treated with 50 μg/mL Tanshinol (*P* > 0.05) when compared to the control cells. Down-regulation of Bax whereas up-regulation of Bcl-2 were found in tanshinol treated cells, and the Bcl-2/Bax ratio were significantly increased (*P* < 0.05; **Fig 2C** and **2D**). Besides, it seems that higher concentration of tanshinol possessed a greater alteration, indicating tanshinol promoted osteoblast viability and ALP activity while reduced apoptosis, all in a dose-dependent manner.

### Tanshinol up-regulated the expression of Col1A1, Runx2 and OCN

**Fig 3:**
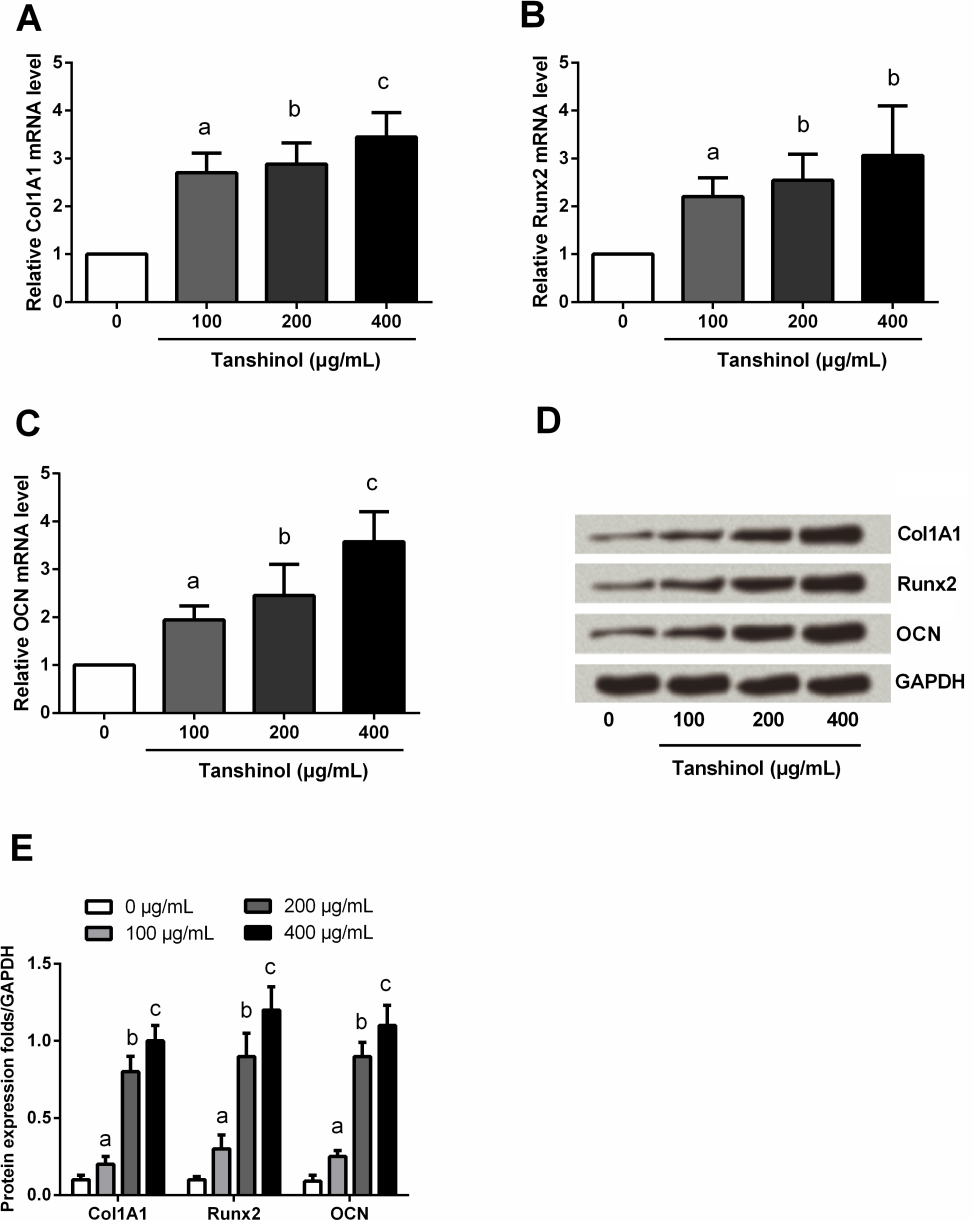
Tanshinol up-regulated the expression of Col1A1, Runx2 and OCN. MC3T3-E1 cells were treated with 0–400 μg/mL tanshinol, and then the mRNA level expressions of (A) Col1A1, (B) Runx2 and (C) OCN were detected by qPCR. (D) and (E) The protein expressions of these three factors were determined by Western blot. Col1A1, Collagen Type I Alpha 1; Runx2, Runt Related Transcription Factor 2; OCN, osteocalcin; qPCR, quantitative PCR; GAPDH, Glyceraldehyde-3-Phosphate Dehydrogenase. n = 3. Different lowercase letters above the columns indicate that the mean values of different groups are significantly different (*P* < 0.05).

Col1A1, Runx2 and OCN are three marker genes of osteoblastic differentiation, to further explore the effects of tanshinol on osteoblastic cells, the expression of these three markers were detected before and after tanshinol treatment. qPCR and Western blot assays (**Fig 3A–3E**) showed that, both the mRNA and protein levels of Col1A1, Runx2 and OCN were significantly up-regulated after tanshinol treatment (*P* < 0.05). In addition, higher concentration of tanshinol had a stronger regulation in the expression of these three factors, indicating tanshinol up-regulated the expression of Col1A1, Runx2 and OCN, also in a dose-dependent manner.

### Tanshinol improved OVX-induced dyslipidemia

**Fig 4:**
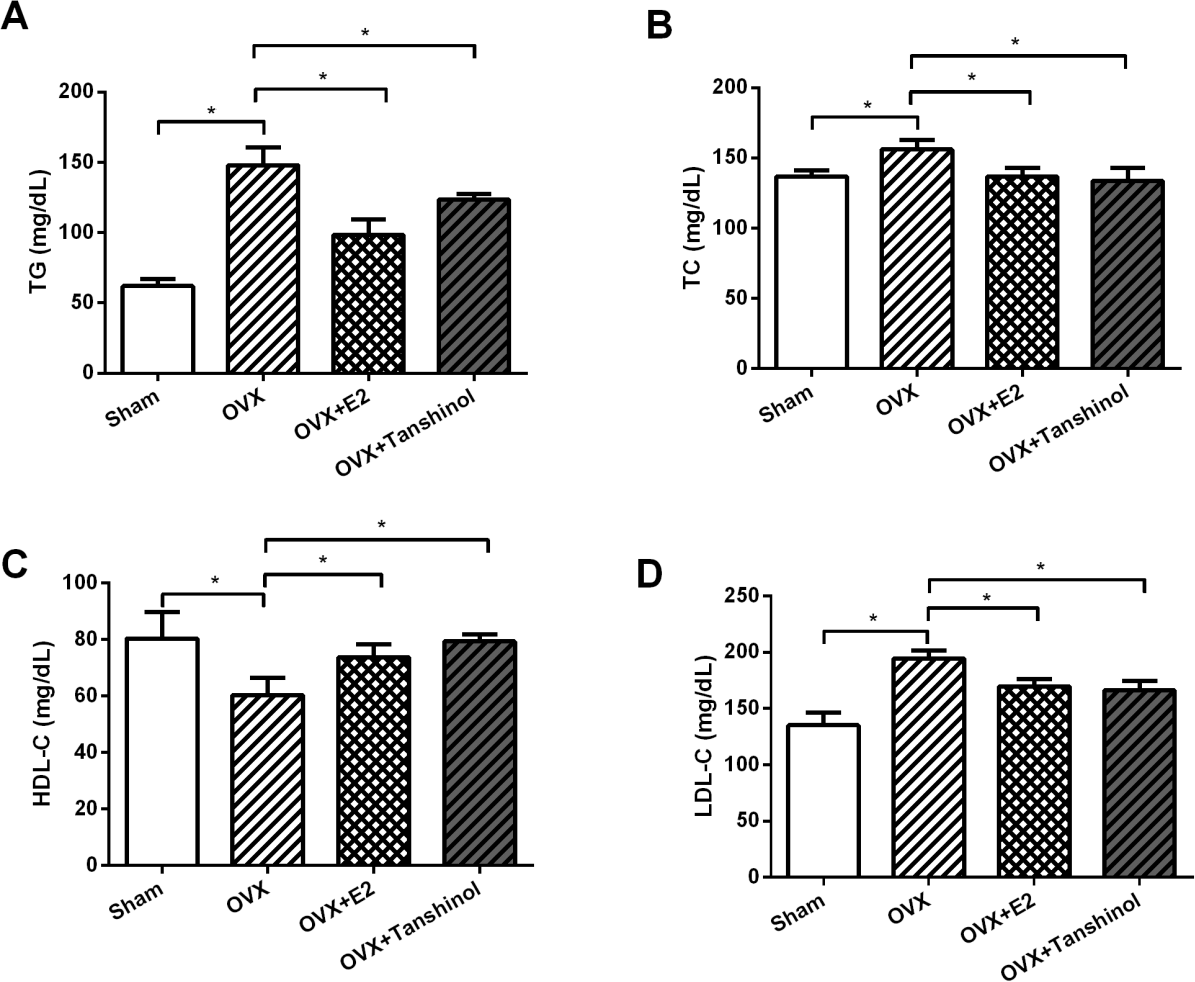
Tanshinol improved OVX-induced dyslipidemia. Rats underwent OVX surgery, tanshinol or E2 treatment, and then (A) TG, (B) TC, (C) HDL-C and (D) LDL-C levels in rats were respectively detected by commercial kits. OVX, ovariectomized; E2, β-estradiol; TG, triglycerides; TC, total cholesterol; HDL-C, high-density lipoprotein cholesterol; LDL-C, low-density lipoprotein cholesterol. n = 3. *, *P* < 0.05.

Next, we explored the functions of tanshinol *in vivo* by detection of TG, TC, HDL-C and LDL-C levels in rats which underwent OVX operation, E2 or tanshinol treatment. We found that (**Fig 4A–4D**), TG, TC and LDL-C were all increased by OVX, whereas HDL-C was decreased by OVX (*P* < 0.05). More important, E2 and tanshinol alleviated the aberrant regulation of these four plasma lipid index induced by OVX (*P* < 0.05).

### Tanshinol reduced OVX-elevated the markers of bone turnover

**Fig 5:**
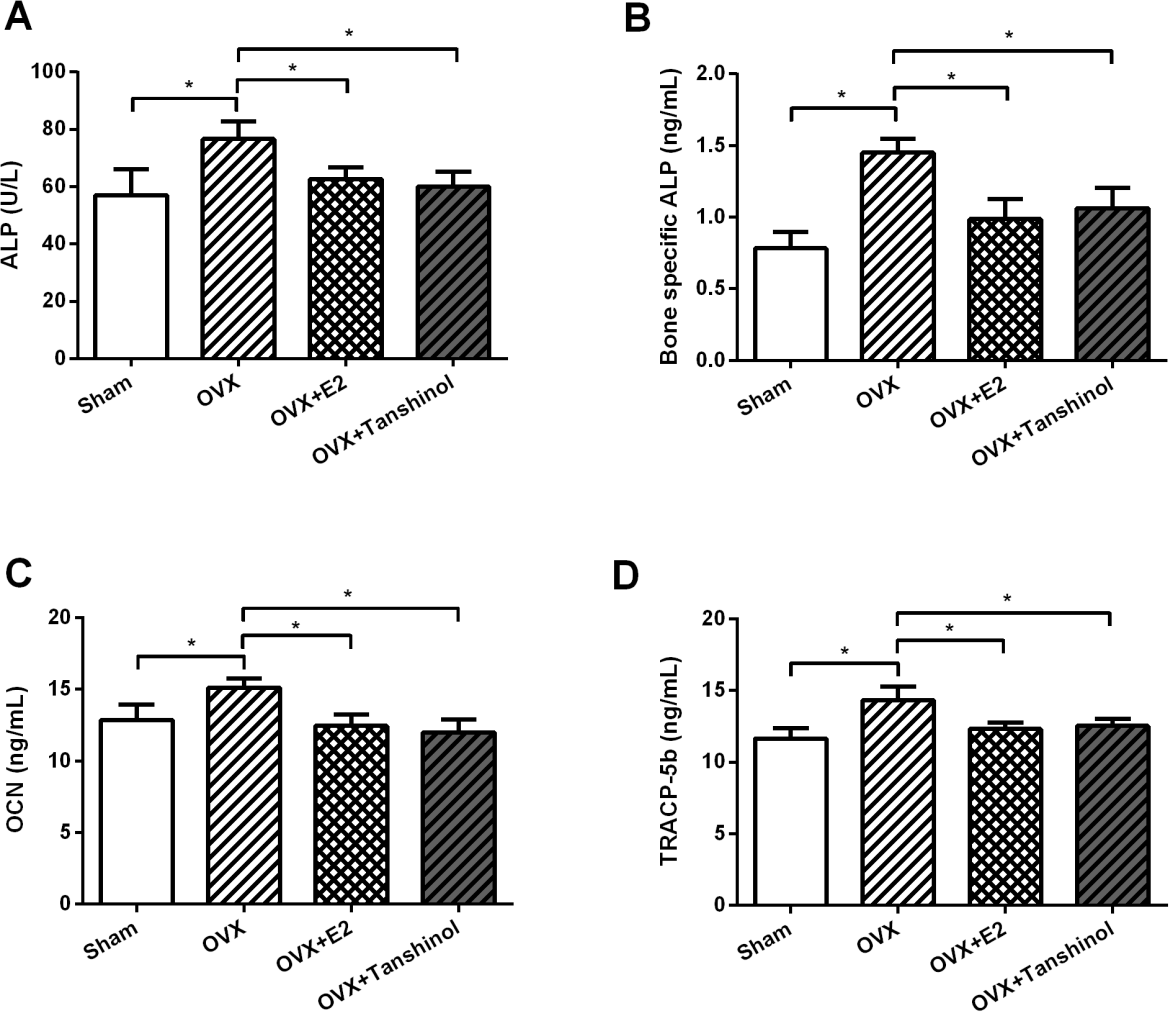
Tanshinol reduced OVX-elevated the markers of bone turnover. Rats underwent OVX surgery, tanshinol or E2 treatment, and then the contents of (A) serum and (B) bone-specific ALP, (C) OCN and (D) TRACP-5b in rats were measured by using commercial kits. OVX, ovariectomized; E2, β-estradiol; ALP, alkaline phosphatase; OCN, osteocalcin; TRACP-5b, Tartrate-resistant acid phosphatase-5b. n = 3. *, *P* < 0.05.

To explore whether tanshinol plays a role in bone metabolism, four metabolic markers of bone turner in rats which underwent OVX operation, E2 or tanshinol treatment were evaluated. The increase of serum and bone-specific ALP, OCN and TRACP-5b were found in OVX rats (*P* < 0.05; **Fig 5A–5D**). Surprisingly, E2 and tanshinol recovered these remarkable increases induced by OVX (*P* < 0.05).

### Tanshinol blocked NF-κB pathway

**Fig 6:**
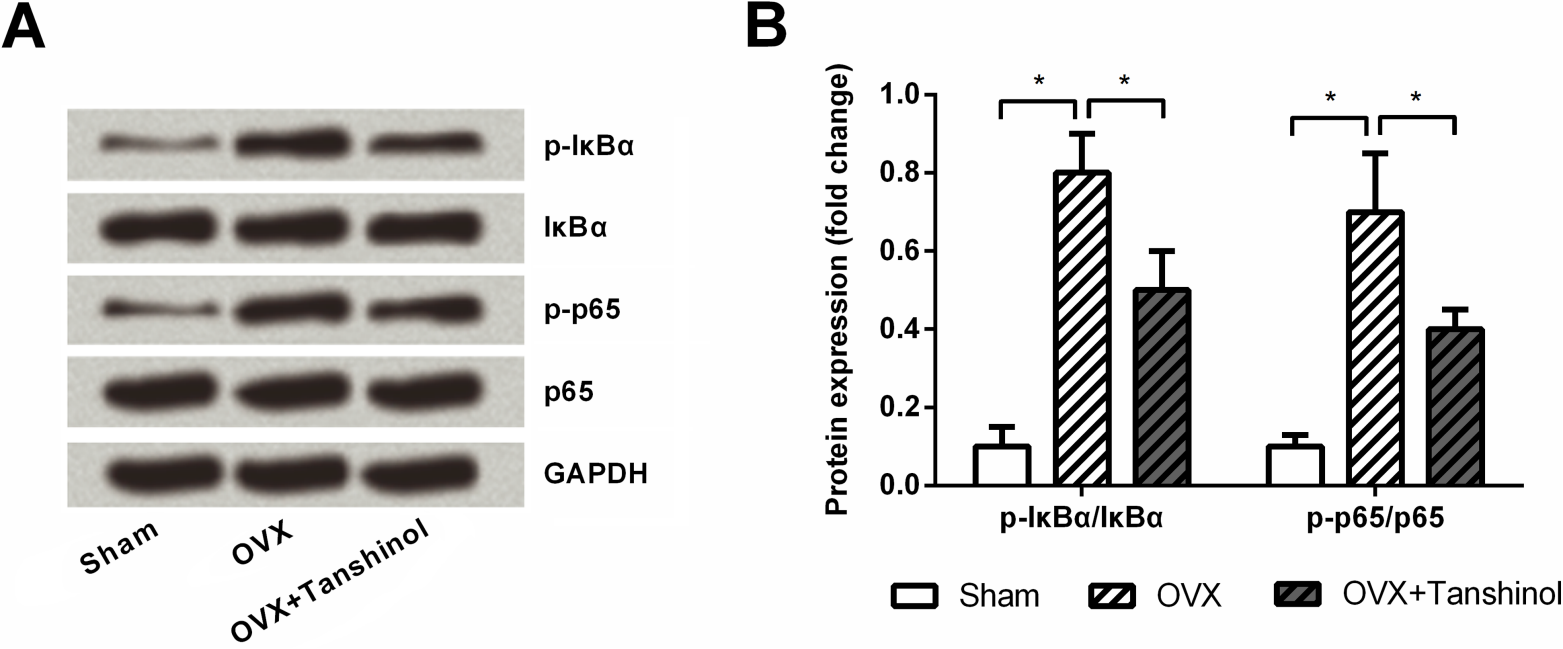
Tanshinol blocked NF-κB pathway. (A) and (B) Rats underwent OVX surgery with or without tanshinol treatment, and then the expressions of main factors, *i*.*e*., IκBα, p-IκBα, p65 and p-p65, in NF-κB pathway in rat metaphyseal and diaphyseal bone were determined by Western blot analysis. OVX, ovariectomized; NF-κB, nuclear factor-kappa B; IκBα, inhibitory subunit of NF-κB; p-IκBα, phosphorylated IκBa; p-p65, phosphorylated p65; GAPDH, Glyceraldehyde-3-Phosphate Dehydrogenase. n = 3. *, *P* < 0.05.

To further explore the possible mechanism in which tanshinol affected OVX-elevated the markers of bone turnover, the expressions of main factors in NF-κB pathway were determined in rats which underwent OVX operation and tanshinol treatment. Western blotting (**Fig 6A** and **6B**) showed that, both p-IκBα and p-p65 were up-regulated by OVX (*P* < 0.05), while the up-regulations were partly abolished by addition of tanshinol (*P* < 0.05). Both OVX and tanshinol had no impacts on the expression of IκBα and p65.

## Discussion

In this study, we found that tanshinol promoted osteoblast viability and ALP activity while reduced apoptosis; additionally, Col1A1, Runx2 and OCN were all up-regulated by tanshinol *in vitro*. *In vivo* investigations, tanshinol alleviated OVX-induced abnormal regulation of TG, TC, HDL-C, LDL-C, ALP, OCN and TRACP-5b. Furthermore, the up-regulation of phosphorylation form of IκBα and p65 in OVX rats were both recovered by tanshinol.

Bone formation is primarily functioned by osteoblast, and the increased proliferation and differentiation of osteoblast can enhance bone formation [16]. Previous study has demonstrated that tanshinol could attenuate suppression of osteoblastic differentiation induced by oxidative stress via Wnt/forkhead box O3A (FoxO3a) signaling pathway [17]. A later study confirmed the notion that tanshinol attenuated the decrease of bone formation and bone mass and bone quality elicited by glucocorticoid [18]. In line with these previous studies, our data suggested that tanshinol might be beneficial for bone formation by promoting osteoblast viability and ALP activity, as well as by suppressing apoptosis. Col1A1, Runx2 and OCN are three osteogenic specific genes which have been identified as osteoblastic markers [19]. Moreover, Runx2 is expressed in the earliest stage of osteogenic differentiation, and can trigger bone extracellular cartilage matrix proteins Col1A1 and OCN [20]. In the present study, both the mRNA and protein levels of these three markers were all up-regulated by tanshinol. These data provided evidence that tanshinol might have regulatory effects on the markers of bone formation.

OVX induces postmenopausal osteoporosis in rats is a commonly used experimental, and this model is characterized by bone loss and an increased bone turnover due to estrogen deficiency [21]. In OVX rats, a significant increase of serum and bone ALP, OCN and TRACP have been recorded [22, 23]. ALP and TRACP are respectively secreted by osteoblasts and osteoclasts, ALP is essential for bone mineralization while TRACP, especially TRACP-5b, is correlates with resorptive activity [24, 25]. It seems that these two factors are associated with bone formation, but the mechanism of this formation is still unclear. OCN is known as serum markers reflecting osteoblast activities including bone formation and turnover [26]. In the current study, tanshinol remarkably recovered OVX-induced up-regulation of ALP, OCN and TRACP-5b, which indicated a protective role of tanshinol in OVX-induced bone turnover. In addition, recent studies demonstrated lipid metabolic disorders with hyperlipidemia and hypercholesterolemia as a distinct risk for osteoporosis in OVX rats [27]. Also, an increase of visceral fat accumulation, TC and LDL-C, as well as a decrease of HDL-C were found after OVX surgery [28]. Interestingly, our data displayed that tanshinol notably recovered the dysregulation of TG, TC, HDL-C and LDL-C induced by OVX, suggesting tanshinol could improve OVX-induced dyslipidemia. Similarly, previous studies have indicated tanshinol exerted a protective action on bone formation in glucocorticoid treated rats by stimulating osteogenesis and depressing adipogenesis [29, 30].

NF-κB pathway plays critical role in many cellular processes, such as cell survival, inflammation and differentiation [31, 32]. NF-κB pathway is activated through degradation of IκBα and then triggering the translocation of various heterodimers, predominantly p65/p50, to the nucleus [33]. Currently, studies have revealed that NF-κB suppressed osteoblast differentiation and ultimately caused bone loss [32]. In an OVX mouse model, the inhibitory function of NF-κB pathway on osteoblast differentiation was uncovered [34]. In the current study, the IκBα and p65 were both remarkably phosphorylated by OVX, while the phosphorylation was partially neutralized by tanshinol. Our data provide the evidence that tanshinol promoted bone formation possibly via blocking NF-κB pathway.

There are several limitations exist in this study. First, we revealed the effects of tanshinol on the immortalized MC3T3-E1 cells, while we did not investigate whether tanshinol also has the similar impacts on primary osteoblast, which may further improve the findings. Second, this study lack of assessment of the phenotype of cultured MC3T3-E1 cells, matrix mineralization should be quantified by performing Alizarin red S staining, which may provide stronger evidence of differentiation. Third, micro-computed tomography for analysis of the trabecular bone mass phenotype of OVX rat was required, to further evidence that tanshinol might have functional effects on bone formation. Fourth, deeper of investigations are needed to scrutinize the underling mechanisms of tanshinol in osteoporosis.

## Conclusions

To sum up, our *in vitro* and *in vivo* investigations indicated tanshinol may has benefical for bone turnover. Tanshinol may exert a bone protective function at least in part via blocking NF-κB pathway. This study will provide new evidence that tanshinol is a potential therapeutic option for the relief of estrogen deficiency-induced osteoporosis.

## Ethics statement

Experiments using rats were performed in accordance with the National Institute of Health Guide for the Care and Use of Laboratory Animals and were approved by Institutional Animal Care and User Committee of The 2nd Affiliated Hospital of Harbin Medical University.

## References

[1] Du L, Nong MN, Zhao JM, Peng XM, Zong SH, Zeng GF. Polygonatum sibiricum polysaccharide inhibits osteoporosis by promoting osteoblast formation and blocking osteoclastogenesis through Wnt/beta-catenin signalling pathway. Sci Rep. 2016;6:32261 Epub 2016/08/25. doi: 10.1038/srep32261 ; PubMed Central PMCID: PMCPmc4995504. 27554324 10.1038/srep32261 PMC4995504

[2] Nik J, Lai PS, Ng CJ, Emmerton L. A qualitative study of community pharmacists' opinions on the provision of osteoporosis disease state management services in Malaysia. BMC health services research. 2016;16:448 Epub 2016/09/01. doi: 10.1186/s12913-016-1686-x . 27577560 10.1186/s12913-016-1686-x PMC5006277

[3] Sinnesael M, Claessens F, Boonen S, Vanderschueren D. Novel insights in the regulation and mechanism of androgen action on bone. Current opinion in endocrinology, diabetes, and obesity. 2013;20(3):240–4. Epub 2013/03/02. doi: 10.1097/MED.0b013e32835f7d04 . 23449008 10.1097/MED.0b013e32835f7d04

[4] Pan XW, Zhao XH. In Vitro Proliferation and Anti-Apoptosis of the Papain-Generated Casein and Soy Protein Hydrolysates towards Osteoblastic Cells (hFOB1.19). Int J Mol Sci. 2015;16(6):13908–20. Epub 2015/06/20. doi: 10.3390/ijms160613908 ; PubMed Central PMCID: PMCPmc4490530. 26090716 10.3390/ijms160613908 PMC4490530

[5] Park MH, Kim S, Cheon J, Lee J, Kim BK, Lee SH, et al Effects of Scytosiphon lomentaria on osteoblastic proliferation and differentiation of MC3T3-E1 cells. Nutrition research and practice. 2016;10(2):148–53. Epub 2016/04/19. doi: 10.4162/nrp.2016.10.2.148 ; PubMed Central PMCID: PMCPmc4819124. 27087897 10.4162/nrp.2016.10.2.148 PMC4819124

[6] Luo S, Yang Y, Chen J, Zhong Z, Huang H, Zhang J, et al Tanshinol stimulates bone formation and attenuates dexamethasone-induced inhibition of osteogenesis in larval zebrafish. Journal of Orthopaedic Translation. 2015;4:35–45. 10.1016/j.jot.2015.07.002 PMC5986998 30035064

[7] Chong CM, Zhou ZY, Razmovski-Naumovski V, Cui GZ, Zhang LQ, Sa F, et al Danshensu protects against 6-hydroxydopamine-induced damage of PC12 cells in vitro and dopaminergic neurons in zebrafish. Neuroscience letters. 2013;543:121–5. Epub 2013/04/09. doi: 10.1016/j.neulet.2013.02.069 . 23562886 10.1016/j.neulet.2013.02.069

[8] Li J, He C, Tong W, Zou Y, Li D, Zhang C, et al Tanshinone IIA blocks dexamethasone-induced apoptosis in osteoblasts through inhibiting Nox4-derived ROS production. Int J Clin Exp Pathol. 2015;8(10):13695–706. Epub 2016/01/02. ; PubMed Central PMCID: PMCPmc4680542. 26722597 PMC4680542

[9] Gu X, Han D, Chen W, Zhang L, Lin Q, Gao J, et al SIRT1-mediated FoxOs pathways protect against apoptosis by promoting autophagy in osteoblast-like MC3T3-E1 cells exposed to sodium fluoride. Oncotarget. 2016 Epub 2016/08/27. doi: 10.18632/oncotarget.11573 . 27564107 10.18632/oncotarget.11573 PMC5323150

[10] Livak KJ, Schmittgen TD. Analysis of relative gene expression data using real-time quantitative PCR and the 2(-Delta Delta C(T)) Method. Methods. 2001;25(4):402–8. Epub 2002/02/16. doi: 10.1006/meth.2001.1262 . 11846609 10.1006/meth.2001.1262

[11] Choi JH, Kim TS, Park JK, Sim YJ, Kim K, Lee SJ. Short-term treadmill exercise preserves sensory-motor function through inhibiting apoptosis in the hippocampus of hypoxic ischemia injury rat pups. Journal of exercise rehabilitation. 2013;9(5):457–62. Epub 2013/11/28. doi: 10.12965/jer.130055 ; PubMed Central PMCID: PMCPmc3836552. 24282805 10.12965/jer.130055 PMC3836552

[12] Ngo Sock ET, Mayer G, Lavoie JM. Combined Effects of Rosuvastatin and Exercise on Gene Expression of Key Molecules Involved in Cholesterol Metabolism in Ovariectomized Rats. PLoS One. 2016;11(7):e0159550 Epub 2016/07/22. doi: 10.1371/journal.pone.0159550 ; PubMed Central PMCID: PMCPmc4956224. 27442011 10.1371/journal.pone.0159550 PMC4956224

[13] Zhao X, Wu ZX, Zhang Y, Yan YB, He Q, Cao PC, et al Anti-osteoporosis activity of Cibotium barometz extract on ovariectomy-induced bone loss in rats. Journal of ethnopharmacology. 2011;137(3):1083–8. Epub 2011/07/26. doi: 10.1016/j.jep.2011.07.017 . 21782010 10.1016/j.jep.2011.07.017

[14] Jeon EJ, Lee DH, Kim YJ, Ahn J, Kim MJ, Hwang JT, et al Effects of yuja peel extract and its flavanones on osteopenia in ovariectomized rats and osteoblast differentiation. Molecular nutrition & food research. 2016 Epub 2016/08/11. doi: 10.1002/mnfr.201600257 . 27506630 10.1002/mnfr.201600257

[15] Schreiweis MA, Butler JP, Kulkarni NH, Knierman MD, Higgs RE, Halladay DL, et al A proteomic analysis of adult rat bone reveals the presence of cartilage/chondrocyte markers. J Cell Biochem. 2007;101(2):466–76. Epub 2007/01/06. doi: 10.1002/jcb.21196 . 17205546 10.1002/jcb.21196

[16] Lane NE, Kelman A. A review of anabolic therapies for osteoporosis. Arthritis research & therapy. 2003;5(5):214–22. Epub 2003/08/23. doi: 10.1186/ar797 ; PubMed Central PMCID: PMCPmc193734. 12932280 10.1186/ar797 PMC193734

[17] Yang Y, Su Y, Wang D, Chen Y, Wu T, Li G, et al Tanshinol attenuates the deleterious effects of oxidative stress on osteoblastic differentiation via Wnt/FoxO3a signaling. Oxidative medicine and cellular longevity. 2013;2013:351895 Epub 2014/02/04. doi: 10.1155/2013/351895 ; PubMed Central PMCID: PMCPmc3893867. 24489983 10.1155/2013/351895 PMC3893867

[18] Yang Y, Su Y, Wang D, Chen Y, Liu Y, Luo S, et al Tanshinol Rescues the Impaired Bone Formation Elicited by Glucocorticoid Involved in KLF15 Pathway. 2016;2016:1092746 doi: 10.1155/2016/1092746 . 27051474 10.1155/2016/1092746 PMC4808655

[19] Jin P, Liao L, Lin X, Guo Q, Lin C, Wu H, et al Stimulating effect of a novel synthesized sulfonamido-based gallate ZXHA-TC on primary osteoblasts. Yonsei medical journal. 2015;56(3):760–71. Epub 2015/04/04. doi: 10.3349/ymj.2015.56.3.760 ; PubMed Central PMCID: PMCPmc4397447. 25837183 10.3349/ymj.2015.56.3.760 PMC4397447

[20] Komori T. Regulation of bone development and maintenance by Runx2. Frontiers in bioscience: a journal and virtual library. 2008;13:898–903. Epub 2007/11/06. . 17981598 10.2741/2730

[21] Kalu DN. The ovariectomized rat model of postmenopausal bone loss. Bone and mineral. 1991;15(3):175–91. Epub 1991/12/01. . 1773131 10.1016/0169-6009(91)90124-i

[22] Abuohashish HM, Ahmed MM, Al-Rejaie SS, Eltahir KE. The antidepressant bupropion exerts alleviating properties in an ovariectomized osteoporotic rat model. Acta pharmacologica Sinica. 2015;36(2):209–20. Epub 2014/12/30. doi: 10.1038/aps.2014.111 ; PubMed Central PMCID: PMCPmc4326785. 25544359 10.1038/aps.2014.111 PMC4326785

[23] Hassan HA, El Wakf AM, El Gharib NE. Role of phytoestrogenic oils in alleviating osteoporosis associated with ovariectomy in rats. Cytotechnology. 2013;65(4):609–19. Epub 2012/11/20. doi: 10.1007/s10616-012-9514-6 ; PubMed Central PMCID: PMCPmc3720960. 23161222 10.1007/s10616-012-9514-6 PMC3720960

[24] Havill LM, Hale LG, Newman DE, Witte SM, Mahaney MC. Bone ALP and OC reference standards in adult baboons (Papio hamadryas) by sex and age. Journal of medical primatology. 2006;35(2):97–105. Epub 2006/03/25. doi: 10.1111/j.1600-0684.2006.00150.x . 16556296 10.1111/j.1600-0684.2006.00150.x

[25] Zhang DW, Wang ZL, Qi W, Zhao GY. The effects of Cordyceps sinensis phytoestrogen on estrogen deficiency-induced osteoporosis in ovariectomized rats. BMC Complement Altern Med. 2014;14:484 Epub 2014/12/17. doi: 10.1186/1472-6882-14-484 ; PubMed Central PMCID: PMCPmc4302055. 25496560 10.1186/1472-6882-14-484 PMC4302055

[26] Polak-Jonkisz D, Zwolinska D. Osteocalcin as a biochemical marker of bone turnover. Nephrology. 1998;4(5–6):339–46.

[27] Hassan HA, Abdel-Wahhab MA. Effect of soybean oil on atherogenic metabolic risks associated with estrogen deficiency in ovariectomized rats: dietary soybean oil modulate atherogenic risks in overiectomized rats. Journal of physiology and biochemistry. 2012;68(2):247–53. Epub 2011/12/30. doi: 10.1007/s13105-011-0137-8 . 22205583 10.1007/s13105-011-0137-8

[28] Deshaies Y, Dagnault A, Lalonde J, Richard D. Interaction of corticosterone and gonadal steroids on lipid deposition in the female rat. The American journal of physiology. 1997;273(2 Pt 1):E355–62. Epub 1997/08/01. . 9277389 10.1152/ajpendo.1997.273.2.E355

[29] Cui L, Liu YY, Wu T, Ai CM, Chen HQ. Osteogenic effects of D+beta-3,4-dihydroxyphenyl lactic acid (salvianic acid A, SAA) on osteoblasts and bone marrow stromal cells of intact and prednisone-treated rats. Acta pharmacologica Sinica. 2009;30(3):321–32. Epub 2009/03/06. doi: 10.1038/aps.2009.9 ; PubMed Central PMCID: PMCPmc4002398. 19262556 10.1038/aps.2009.9 PMC4002398

[30] Cui L, Li T, Liu Y, Zhou L, Li P, Xu B, et al Salvianolic acid B prevents bone loss in prednisone-treated rats through stimulation of osteogenesis and bone marrow angiogenesis. PLoS One. 2012;7(4):e34647 Epub 2012/04/12. doi: 10.1371/journal.pone.0034647 ; PubMed Central PMCID: PMCPmc3321026. 22493705 10.1371/journal.pone.0034647 PMC3321026

[31] Cieniewicz B, Santana AL, Minkah N, Krug LT. Interplay of Murine Gammaherpesvirus 68 with NF-kappaB Signaling of the Host. Frontiers in microbiology. 2016;7:1202 Epub 2016/09/02. doi: 10.3389/fmicb.2016.01202 ; PubMed Central PMCID: PMCPmc4987367. 27582728 10.3389/fmicb.2016.01202 PMC4987367

[32] Li A, Yang L, Geng X, Peng X, Lu T, Deng Y, et al Rocaglamide-A Potentiates Osteoblast Differentiation by Inhibiting NF-kappaB Signaling. Molecules and cells. 2015;38(11):941–9. Epub 2015/11/10. doi: 10.14348/molcells.2015.2353 ; PubMed Central PMCID: PMCPmc4673408. 26549505 10.14348/molcells.2015.2353 PMC4673408

[33] Maruyama W, Shirakawa K, Matsui H, Matsumoto T, Yamazaki H, Sarca AD, et al Classical NF-kappaB pathway is responsible for APOBEC3B expression in cancer cells. Biochem Biophys Res Commun. 2016 Epub 2016/09/01. doi: 10.1016/j.bbrc.2016.08.148 . 27577680 10.1016/j.bbrc.2016.08.148

[34] Chang J, Wang Z, Tang E, Fan Z, McCauley L, Franceschi R, et al Inhibition of osteoblastic bone formation by nuclear factor-kappaB. Nat Med. 2009;15(6):682–9. Epub 2009/05/19. doi: 10.1038/nm.1954 ; PubMed Central PMCID: PMCPmc2768554. 19448637 10.1038/nm.1954 PMC2768554

